# A filled duration illusion in music: Effects of metrical subdivision on the perception and production of beat tempo.

**DOI:** 10.2478/v10053-008-0071-7

**Published:** 2010-01-13

**Authors:** Bruno H. Repp, Meijin Bruttomesso

**Affiliations:** 1 Haskins Laboratories, New Haven, Connecticut; 2 Yale University, New Haven, Connecticut

**Keywords:** timing, tempo perception, interval subdivision, filled duration illusion, music performance

## Abstract

This study replicates and extends previous findings suggesting that metrical subdivision slows the perceived beat tempo (Repp, 2008). Here, musically trained participants produced the subdivisions themselves and were found to speed up, thus compensating for the perceived slowing. This was shown in a synchronization-continuation paradigm (Experiment 1) and in a reproduction task (Experiment 2a). Participants also judged the tempo of a subdivided sequence as being slower than that of a preceding simple beat sequence (Experiment 2b). Experiment 2 also included nonmusician participants, with similar results. Tempo measurements of famous pianists’ recordings of two variation movements from Beethoven sonatas revealed a strong tendency to play the first variation (subdivided beats) faster than the theme (mostly simple beats). A similar tendency was found in musicians’ laboratory performances of a simple theme and variations, despite instruc-tions to keep the tempo constant (Experiment 3a). When playing melodic sequences in which only one of three beats per measure was subdivided, musicians tended to play these beats faster and to perceive them as longer than adjacent beats, and they played the whole sequence faster than a sequence without any subdivisions (Experiments 3b and 3c). The results amply demonstrate a *filled duration illusion* in rhythm perception and music performance: Intervals containing events seem longer than empty intervals and thus must be shortened to be perceived as equal in duration.

##  

“But what they did to me was give me a metronome and a theme which you play in quicker and quicker note values: triplets, eighths, sixteenths, and so on. I know that orchestras, when they see a lot of black notes, usually start to accelerate. I made, I think, a 2 per cent or 3 per cent error over the whole test. So they said, ‘Herr von Karajan apparently has a computer in his brain!’“ R. Osborne (1989, p. 97)^1^

## INTRODUCTION

The present study investigates whether certain findings on the perception of temporal interval duration generalize to rhythmic sequences of intervals and thus are relevant to music perception and performance. Psychophysical research on duration perception has repeatedly shown that filled auditory intervals are perceived as longer than empty intervals (Adams, 1977; Buffardi, 1971; Craig, 1973; Goldfarb & Goldstone, 1963; Hall & Jastrow, 1886; Ornstein, 1969; Thomas & Brown, 1974). This filled duration illusion (FDI) is particularly large when a continuous tone is compared with a silent interval (Craig, 1973; Wearden, Norton, Martin, & Montford-Bebb, 2007), but it is also evident when discrete events are inserted into a silent interval (Nakajima, 1979, 1987; Thomas & Brown, 1974).^2^ In some studies, these interval subdivisions were equally spaced (Buffardi, 1971; Grimm, 1934; Thomas & Brown, 1974), which approximates a metrical musical rhythm. On the whole, however, this perceptual research was not concerned with music and often used only single intervals.

In a study of motor timing, Wohlschläger and Koch (2000) used interval subdivision to address the *negative mean asynchrony* in sensorimotor synchronization: When participants tap in synchrony with a simple auditory beat (an isochronous sequence of identical tones), their taps typically precede the tones by some tens of milliseconds, on average. Wohlschläger and Koch proposed that this could be explained by a perceptual underestimation of the duration of the empty intervals between beats. They tested this hypothesis by inserting soft clicks (“raindrops”) at random times into the inter-beat intervals (IBIs) or by asking participants to carry out an additional movement during the IBIs. This indeed reduced or eliminated the negative mean asynchrony. The inserted sounds or movements thus seemed to reduce the underestimation of interval duration, which is consistent with the literature on the FDI. Because this research involved tapping in synchrony with a beat, it seems relevant to music, but the randomly timed raindrops were not particularly musical.

In a recent series of experiments, Repp (2008) demonstrated an effect of metrical (i.e., regularly spaced) subdivision of IBIs on the perception and production of beat tempo. *Beat tempo* is the rate of the events that function as main beats of a rhythm, which naturally reflects the duration of the IBIs. The purpose of Repp’s research was to test whether metrical subdivision of a beat sequence would cause an FDI (i.e., make the IBIs seem longer and the beat tempo slower) even when the participants are musically trained and thus experts in tempo perception and timing control. He used three different tasks: synchronization-continuation tapping, reproduction of a sequence by tapping, and perceptual judgment. In the first task, participants tapped in synchrony with an isochronous auditory beat that was either simple or subdivided (by one, two, or three additional tones) and continued tapping the beat after the sequence stopped. The results revealed that all participants tapped slower when continuing a subdivided beat than when continuing a simple beat, in accord with the FDI hypothesis.^3^ In the reproduction task, participants listened to a short target sequence of either simple or subdivided beats (two subdivision tones per IBI) and then reproduced the beats of that sequence after a pause, attempting to match the target tempo with their taps. As expected, the musicians were quite accurate in the reproduction of simple beats, but they tapped too slowly when reproducing subdivided beats. In the perceptual judgment task, participants were presented with a simple or subdivided standard sequence that was followed by a slower, equal, or faster comparison sequence of simple beats. As predicted, the comparison sequence had to be slower in order to be judged as having the same tempo as a subdivided standard sequence. The study included some additional variants of the synchronization-continuation tapping and perceptual judgment tasks, with largely congruent results. (One exception will be mentioned later.) Also, a small group of nonmusicians was tested, who showed larger subdivision effects than the musicians in the reproduction task, but (surprisingly) smaller effects in the perceptual judgment task. Overall, the findings demonstrated that sequences whose IBIs are metrically subdivided are perceived as having a slower beat tempo than those that are not subdivided. In other words, a FDI did occur in metrical contexts and with musician participants, although the effect was relatively small (about 3% of the IBI duration).^4^

The purpose of the present investigation was to complement and extend the research just summarized. In the previous study, with the exception of some special conditions in Experiment 2, subdivided sequences always occurred first in each task. This had the advantage that, when required to continue or reproduce a simple or subdivided beat, participants only had to tap a simple beat, so that difficulties of motor execution could not play a role. However, it created an imbalance in the design. Moreover, in musical contexts the reverse order is more common. For example, in compositions with theme and variations, a simple theme generally precedes more complex variations. Also, previous studies of the FDI have found effects of order of presentation; in particular, the FDI was reduced or absent when the silent interval preceded the filled interval (Grimm, 1934; Hall & Jastrow, 1886; Meumann, 1896; Nakajima, 1979, 1987). Therefore, Experiments 1 and 2 of the present study attempted to replicate the findings of Repp (2008) using the same three tasks, but with a reversed order of sequences. In synchronization-continuation and reproduction, this means that participants had to tap either simple or subdivided sequences, producing the subdivisions themselves with the other hand.^5^ Because a subdivided sequence is potentially more difficult to execute than a simple sequence, subdivision might slow the tapping tempo. However, the FDI hypothesis predicts that a subdivided sequence should be produced at a faster tempo than a simple sequence, in order to be perceived as having the same tempo. In other words, participants need to compensate for the perceived slower tempo of a subdivided sequence by speeding up. Therefore, although the confounding of subdivision with motor difficulty could potentially obscure the predicted perceptual effect of subdivision, it was not a serious concern, especially in musicians who are manually skilled.

Following Experiments 1 and 2, some rough measurements of commercially recorded music performances were conducted, to see whether pianists tend to play a variation faster than the preceding theme, as the FDI hypothesis predicts. This was then followed up in the laboratory with performances of a very simple composition consisting of a theme and variations (Experiment 3a). Experiments 3b and 3c extended the research further to sequences in which only some IBIs are subdivided, to see whether the FDI can have local timing effects on performance and perception.

## EXPERIMENT 1: Synchronization-Continuation

Experiment 1 was modeled after the “baseline” condition of Experiment 2 of Repp (2008), which in turn was a reduced version of Experiment 1 in that study. In these previous experiments, participants tapped in synchrony with a simple or subdivided beat and then continued to tap the simple beat. They were found to tap slower when continuing a subdivided beat than when continuing a simple beat. In the present experiment, they tapped in synchrony with a simple beat and then continued to tap the beat with or without subdivisions. The FDI hypothesis predicts that they should tap faster when tapping a subdivided beat than when tapping a simple beat, so as to compensate for the perceived slower tempo of the former.

### Methods

#### Participants

Eight paid volunteers and both authors (B.H.R., M.B.) participated. The former (ages 22-28, 4 men and 4 women) were graduate students at the Yale School of Music (2 pianists, 3 clarinetists, 1 oboist, 1 cellist, and 1 harpist) who had studied their primary instrument intensively for 13-21 years. B.H.R. (age 63, male) has had 10 years of piano instruction as a child and has played ever since at an advanced amateur level, and M.B. (age 21, female) also has substantial music training (8 years of violin, 5 years of piano, 2 years of bass guitar).

#### Materials and equipment

Each auditory pacing sequence during synchronization contained 12 beats.^6^ The first 10 beats were represented by high-pitched digital piano tones (A7, 3520 Hz). The last two beat tones were lower in pitch (by 3 and 5 semitones, respectively), to signal the end of the pacing sequence. The tones had no specified offset and decayed freely within about 100 ms. Nine different sequences resulted from the crossing of three IBI durations with three subdivision conditions. The IBI durations were 800, 900, and 1000 ms. The subdivision conditions were no subdivision (sub-0), one subdivision (sub-1), and two subdivisions (sub-2). Subdivision tones were 3 semitones lower than beat tones and about 3 dB (10 MIDI velocity units) softer. The initial two IBIs of the pacing sequence were subdivided metrically when subdivisions were required during continuation tapping; this served as an instruction to the participant. Each pacing sequence was followed by a silent interval for continuation tapping that lasted 10 times the IBI duration and was terminated by a single low tone. The participant’s continuation taps produced beat and subdivision tones like those in the pacing sequence.^7^ The nine pacing sequences (3 durations x 3 subdivision conditions) were arranged into 10 random orders (blocks).

The sequences were played on a Roland RD-250s digital piano under control of a program written in MAX 4.0.9. The software ran on an Intel iMac computer that was connected to the digital piano via a MOTU Fastlane-USB MIDI translator. Participants listened to the sequences over Sennheiser HD540 reference II earphones at a comfortable level and tapped with their index fingers on the upper left and upper right segments of a Roland SPD-6 percussion pad held on their lap.

#### Procedure

**Figure 1.**
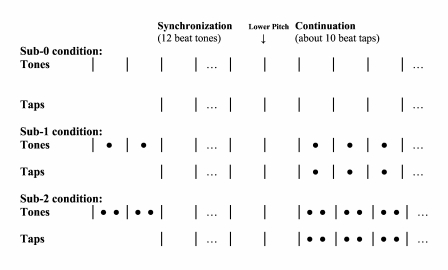
Schematic illustration of the sub-0, sub-1, and sub-2 conditions in Experiment 1. | = pacing beat tone or beat tap; • = subdivision tone or subdivision tap; | = feedback beat tone; • = feedback subdivision tone.

Participants were seated in front of a computer monitor that displayed the current trial number. They were free to adopt their most comfortable style of tapping. They started each trial by pressing the space bar on the computer keyboard. The pacing sequence started 2 s later. Participants were instructed to start tapping with the third beat and to tap in synchrony with the beat with their right hand until the two lower-pitched beats indicated the end of the sequence. If the initial two beats of the pacing sequence were not subdivided, participants were to continue tapping the simple beat with their right hand without interruption. If the initial two beats were subdivided, participants were to continue tapping the beat with their right hand and also tap the appropriate number of subdivisions (one or two per IBI) with their left hand, until the signal to stop tapping sounded. The importance of keeping the beat tempo was emphasized. At the end of each block, participants saved their data and selected the next block. The session lasted approximately 45 min. Figure 1 gives a schematic illustration of the three subdivision conditions.

### Results

**Figure 2.**
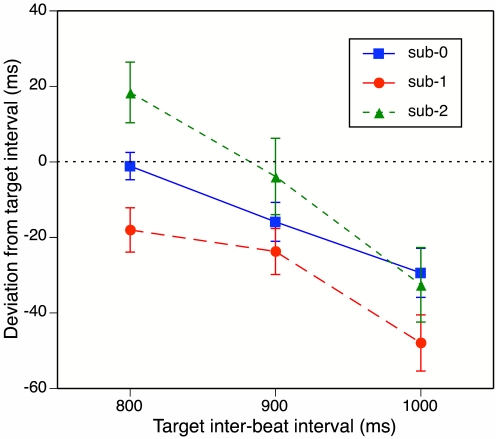
Results of Experiment 1: Mean deviation of the continuation inter-beat interval (IBI) from the target IBI in the three subdivision conditions (sub-0, sub-1, sub-2) as a function of target IBI duration. The dotted horizontal line represents what exact continuation would look like. Error bars represent between-participant standard errors.

Naturally, the mean continuation IBI was expected to increase with the IBI duration of the pacing sequence. The dependent variable of primary interest was the deviation of the mean continuation IBI from the target IBI. The mean continuation IBI was computed across seven consecutive right-hand inter-tap intervals, starting with the interval between the second and third continuation taps.^8^ Figure 2 shows these deviations as a function of IBI duration and subdivision condition.

In the baseline (sub-0) condition, participants were very accurate in continuing the beats with a target IBI of 800 ms, but they tapped increasingly too fast as the target IBI increased. As predicted, continuation tapping was faster in the sub-1 condition than in the baseline condition for all three target IBIs. Contrary to predictions, however, continuation tapping in the sub-2 condition was slower than in the baseline condition at the two shorter target IBIs, and about equally fast at the longest IBI.

A repeated-measures analysis of variance (ANOVA) was conducted on the deviation data with the variables of IBI duration (three levels) and subdivision condition (three levels). The main effect of subdivision did not reach significance, *F*(2, 18) = 3.7, *p* = .07. However, the main effect of IBI duration, *F*(2, 18) = 33.2, *p* < .001, and the interaction, *F*(4, 36) = 7.2, *p* = .001, were both very reliable.^9^ To clarify the interaction, the sub-1 and sub-2 conditions were compared to the sub-0 condition in separate ANOVAs. In the analysis comparing sub-0 and sub-1, the main effect of subdivision did not reach significance, *F*(1, 9) = 4.3, *p* = .07. It was apparent that this was due to one participant (a clarinetist) who showed a reversed effect. When this participant’s data were excluded, the subdivision effect was significant, *F*(1, 8) = 12.3, *p* = .008, which indicates that the majority of participants did show the predicted effect.^10^ The main effect of IBI duration was also significant, *F*(2, 16) = 16.4, *p* = .001, but the interaction was not, *F*(2, 16) = 3.4, *p* = .09. In the analysis comparing sub-0 and sub-2, the main effect of subdivision was not significant, *F*(2, 18) = 0.7, *p* = .44, but the main effect of IBI duration, *F*(2, 18) = 45.4, p < .001, and the interaction, *F*(2, 18) = 10.00, *p* = .003, were both reliable.

The significance of subdivision effects could also be assessed at the individual level by treating trial blocks as independent observations in repeated-measures ANOVAs on each participant’s data. Comparing sub-0 and sub-1, seven of ten participants showed the predicted effect of subdivision (three *p* < .001, two *p* < .01, two *p* < .05), two showed no significant effect, and one (aforementioned) showed a reversed effect (*p* < .001). Three also showed an interaction with IBI duration (*p* < .05). Comparing sub-0 and sub-2, only one participant (author M.B.) showed the predicted main effect (*p* = .001), six showed no significant effect, and three showed a reversed effect (one *p* < .001, two *p* < .01). In addition, five participants showed a significant interaction with IBI duration (two p < .01, three *p* < .05).

### Discussion

Experiment 1 was partially successful in demonstrating compensation for the FDI in a synchronization-continuation paradigm. It remains unclear why one participant showed a reversed effect in the sub-1 condition. For the majority of participants, however, the tapping of a single subdivision had the predicted effect of accelerating the beat tempo during continuation tapping. By contrast, the tapping of two subdivisions did not have the predicted effect; on the contrary, it slowed the continuation beat tempo at the shorter target IBIs. Difficulty of execution of two rapid left-hand taps is a possible explanation, as difficulty would tend to increase with tempo. The resulting slowing of beat tempo may have covered up any compensation in tapping for the perceptual effect of subdivision. (However, this explanation is called into question by Experiment 2a; see below.)

Interestingly, Repp (2008, Experiment 2, Condition 4) obtained a similarly anomalous result in a sub-2 synchronization-continuation tapping condition. In that condition, however, participants first tapped the subdivisions while synchronizing with a simple beat and then continued tapping just the beat. In that paradigm, a slowing of continuation tapping was predicted, but participants tapped faster than expected, especially at the longer IBIs (900 and 1000 ms). That finding obviously cannot be attributed to execution difficulty. Moreover, it occurred only when the taps were accompanied by feedback tones, as in the present study. This points towards a perceptual explanation. Indeed, the results for the sub-0 and sub-2 conditions in that previous experiment are strikingly similar to the present results, but the difference in paradigms makes them difficult to reconcile. This remains a mystery to be resolved, but it will not be addressed further here.

## EXPERIMENT 2a: Reproduction

Experiments 2a and 2b were run in the same session in counterbalanced order but are reported separately. In these experiments we attempted to demonstrate a compensatory subdivision effect in matched reproduction and perceptual judgment tasks, as used previously by Repp (2008, Experiment 4), but with reversed roles of simple and subdivided sequences. The matched ranges of IBI durations made it possible to compare results directly, both between experiments and between studies. Moreover, we included both musician and nonmusician participants. Repp had done the same and had found a curious dissociation between the two tasks in nonmusicians: Whereas they showed very large subdivision effects in reproduction, their perceptual effects were small and nonsignificant overall. We wondered whether we could replicate this finding.

Experiment 2a used the reproduction task. Repp (2008, Experiment 4) presented a target sequence with or without subdivision of the beat, and participants were required to reproduce only the beat by tapping. They tapped slower when reproducing a subdivided beat. In the present experiment, the target sequence was never subdivided, and instead participants were instructed to subdivide or not subdivide the reproduced beat by tapping with their other hand. We expected that participants would tap faster when instructed to subdivide, in order to compensate for the perceived slower tempo of their reproduction.

### Methods

#### Participants

The 10 musician participants were the same as in Experiment 1. In addition, 12 nonmusicians (5 men, 7 women, ages 19-25 years) participated. They had responded to a notice posted on Yale campus and were paid for their services. Nine of them had no musical training whatsoever; the other three had had 0.5, 2, and 3 years of lessons, respectively, but had long been musically inactive.

#### Materials

Each trial presented a target sequence consisting of five isochronous beat tones at one of seven IBIs: 660, 690, 720, 750, 780, 810, and 840 ms. The tones were the same as those in Experiment 1. Eight blocks of 14 randomly ordered trials were presented in which each IBI occurred once in each of two subdivision conditions. For musicians, the conditions were sub-0 and sub-2; for nonmusicians, because we thought they might have difficulties with triple subdivision, the conditions were sub-0 and sub-1.^11^

#### Procedure

**Figure 3.**
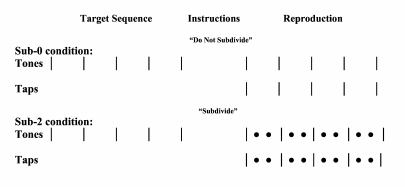
Schematic illustration of the sub-0 and sub-2 conditions in Experiment 2a. | = target beat tone or beat tap; • = subdivision tap; | = feedback beat tone; • = feedback subdivision tone.

Participants started each trial by pressing the space bar on the computer keyboard. The target sequence started 2 s later. Together with the last target tone of each trial, a message appeared on the monitor that directed participants to “Subdivide” or “Do not subdivide.”^12^ Musicians were instructed to skip one beat (i.e., to pause for approximately two IBIs) before making five beat taps at the correct tempo with the right hand and tapping any subdivisions in between with the left hand. Nonmusicians were just told to leave a brief pause before starting to tap. Both beat taps and subdivision taps triggered feedback tones, as in Experiment 1. The experiment lasted approximately 30 min. The tasks for the musicians are shown schematically in Figure 3.

### Results

**Figure 4.**
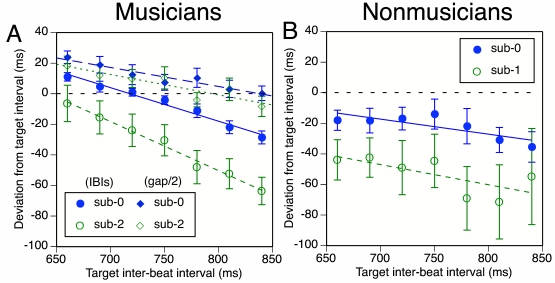
Schematic results of Experiment 2a: Mean deviation of reproduction inter-beat intervals (IBIs) from target IBI duration as a function of target IBI duration in two subdivision conditions, for musicians (circles; sub-0, sub-2) and nonmusicians (sub-0, sub-1). For musicians, results for half the 2-IBI gap between the last target tone and the first reproduction tap are also shown (diamonds). The dotted horizontal line represents what exact reproduction would look like. Error bars represent between-participant standard errors.

As in Experiment 1, the mean reproduction IBI was expected to increase linearly with target IBI duration. The dependent variable of primary interest was the deviation of the mean reproduction IBI from the target IBI. Figure 4 shows these deviations (data symbols: circles) as a function of target IBI duration and subdivision condition.

#### Musicians

In the baseline (sub-0) condition, musicians (Figure 4a) were quite consistent in tapping a bit too slow at the fastest tempo but too fast at the slower tempi, with highest accuracy at a target IBI of 720 ms. When making two subdivision taps during reproduction (sub-2), they tapped faster overall (in contrast to Experiment 1), as predicted by the FDI hypothesis. This acceleration was more pronounced at the slower tempi.

A repeated-measures analysis of variance (ANOVA) was conducted on these data, with the variables of target IBI duration (seven levels) and subdivision condition (two levels). The main effect of subdivision condition was significant, *F*(1, 9) = 9.5, *p* = .013. The analysis also revealed a significant main effect of target IBI duration, *F*(6, 54) = 62.0, *p* < .001, and a significant interaction, *F*(6, 54) = 3.9, *p* = .023.

There were considerable individual differences in the sub-2 condition, as indicated by the error bars. Repeated-measures ANOVAs on individual participants’ data, with trial blocks as the random variable, showed highly reliable effects in the expected direction for six participants (*p* < .001), no significant effect for three participants, and a small reversed effect (*p* < .05) for one participant (the harpist). The clarinetist who had shown reversed subdivision effects in Experiment 1 showed an effect that changed from reversed to predicted as the target IBI increased; this was reflected in a significant interaction (*p* = .005).

The individual subdivision effects (i.e., the differences between the individual mean sub-2 and sub-0 reproduction beat IBIs) were positively correlated with the sub-1 effects in Experiment 1, *r*(8) = .72, *p* < .05, and also tended to be correlated with the sub-2 effects in Experiment 1, *r*(8) = .55, *p* < .10. With the clarinetist outlier omitted, both correlations were significant, *r*(7) = .72, *p* < .05, and .77, p < .01, respectively.

It might be asked whether some compensation for the anticipated perceptual effect of subdivision occurs already during action planning, before tapping has started. Therefore, the “skipped beat” interval between the last target tone and the first reproduction tap was also analyzed. Each such gap was divided in half, and the target IBI duration was subtracted. A few outlier trials, where more than one beat had been skipped, were omitted, and author M.B.’s data were omitted entirely because it emerged that she had not skipped one beat before starting her reproductions. The mean deviations from the target IBI are shown as diamonds in Figure 4a. There was a significant main effect of target IBI duration, *F*(6, 48) = 8.8, *p* = .001, similar to that in continuation tapping but somewhat less pronounced. Interestingly, the main effect of subdivision condition was significant, *F*(1, 8) = 6.4, *p* = .04: Participants started to tap slightly sooner in the sub-2 condition than in the sub-0 condition, perhaps because they already started to subdivide the silent gap in their mind. Clearly, however, this anticipatory subdivision effect was much smaller than the one during reproduction tapping.

Also, the deviations of (half) the gap from the target IBI in the sub-0 condition were generally more positive than the deviations of the IBIs during sub-0 reproduction tapping. This was confirmed in an ANOVA on just these two data sets, *F*(1, 8) = 18.8, *p* = .002. The ANOVA also yielded, in addition to the obvious main effect of IBI duration, *F*(6, 48) = 23.5, *p* < .001, a significant interaction with IBI duration, *F*(6, 48) = 5.4, *p* = .004, which confirms that the deviations during reproduction tapping depended more strongly on IBI duration than the deviations of the “skipped beat” interval did.

#### Nonmusicians

Not unexpectedly, the nonmusicians’ data (Figure 4b) were much more variable than the musicians’. However, all participants were able to perform the task. Nonmusicians generally tapped a bit too fast in the sub-0 condition, but they tapped even faster in the sub-1 condition, as predicted.

In an ANOVA on these data, the effect of subdivision condition was significant, *F*(1, 11) = 5.0, *p* = .047. No other effect was significant. ANOVAs on individual participants’ data revealed that six had a significant subdivision effect in the expected direction (four at *p* < .001, one at *p* < .005, one at p < .05), five showed no reliable effect, and one had a small reversed effect (*p* < .05). The nonmusicians’ gap durations were too inconsistent to be analyzed.

### Discussion

Despite large individual differences, the predicted effect of subdivision did emerge in the reproduction task: Most participants tapped faster beats when they made subdivision taps than when they made none. Remarkably, for musicians this result was obtained in a sub-2 condition, which had not yielded any subdivision effect in Experiment 1. This effectively rules out any explanation of the Experiment 1 results in terms of difficulty of execution, which was not a very plausible explanation to begin with, given the high manual skill of musicians. The task of Experiment 2 differed from that of Experiment 1 in two main respects: Participants started to tap only after the target sequence had ended, and they were required to skip a beat before starting to tap. It is difficult to see why either of these differences should have had such a dramatic effect on the results. There was another difference, however: In Experiment 1, a sub-1 condition occurred in random alternation with sub-2 and sub-0. It could be that this juxtaposition of duple and triple subdivision (sub-1 and sub-2, respectively) introduced a binary bias that had a slowing effect on triple subdivision.

Musicians’ tendency to tap too slow at the faster tempi and too fast at the slower ones is consistent with findings by Jones and McAuley (2005) showing that participants develop a memory of the mean IBI that biases perception or memory of other IBIs. Alternatively, sequential assimilation effects between the IBIs of successive trials could generate a similar regression to the mean. Nonmusicians, however, mainly had a tendency to tap too fast. Musicians also had a bias in that direction, as they did in Experiment 1 (Figure 2).

The finding that (half) the skipped-beat interval produced by musicians was relatively longer than the reproduction IBIs was not predicted but is consistent with the FDI hypothesis. Compared to two successive IBIs, which have a tap in the middle, the skipped-beat interval lacked an explicit subdivision and thus should have been perceived as relatively short. This may have led to compensatory lengthening, so as to retain the feeling of a continuous beat. At the same time, anticipatory mental subdivision led to a slight shortening of the same interval.

Apart from being more variable, the findings for nonmusicians are basically consistent with those for musicians. Thus the present results do not replicate Repp’s (2008) finding of much larger subdivision effects in reproduction for nonmusicians than for musicians (obtained in a sub-3 condition). Several individual nonmusicians, however, did show very large effects indeed, and it is possible that others were slowed down by the requirement of having to make left-hand taps. Of course, comparisons must be made with caution because the nonmusicians had a sub-1 condition, whereas the musicians had a sub-2 condition. Perhaps nonmusicians would have shown larger subdivision effects in a sub-2 condition, but it seems more likely that the difficulty of making two rapid taps with the left hand between right-hand taps might have slowed them down instead. It is also possible that musicians would have shown a smaller subdivision effect in a sub-1 condition, contrary to Experiment 1. Such considerations do not apply to Experiment 2b, however, because there the two participant groups experienced identical conditions (sub-0, sub-2).

## EXPERIMENT 2b: Perceptual judgment

 Experiment 2b was a reversed version of the perceptual task used in Experiment 4 of Repp (2008). Participants heard a standard sequence followed by a comparison sequence and judged their relative tempo. Whereas previously the standard sequence had been either simple or subdivided, it was now the comparison sequence that was either simple or subdivided. Previously, it was found that a simple comparison sequence had to be slower than a subdivided standard sequence to be judged as having the same tempo. Now we predicted that a subdivided comparison sequence would have to be faster than a simple standard sequence to be judged as having the same tempo. 

### Methods

#### Participants

The participants were the same as in Experiment 2a.

#### Materials

Each standard sequence consisted of five isochronous beat tones with an IBI of 750 ms. After a silent interval of 1500 ms, a comparison sequence of five beat tones followed that was either simple (sub-0) or subdivided (sub-2). The comparison sequence IBIs were 660, 690, 720, 750, 780, 810, and 840 ms in duration. (Note that these match the target IBIs in Experiment 2a.) The beat and subdivision tones were the same as in Experiments 1 and 2a. Ten blocks of 14 randomly ordered trials were presented.

#### Procedure

**Figure 5.**
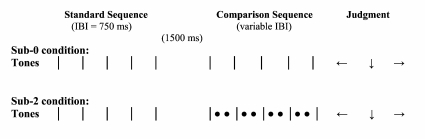
Schematic illustration of the sub-0 and sub-2 conditions in Experiment 2b. | = beat tone; • = subdivision tone; ← = slower, ↓ = same, → = faster.

Participants started the first trial in a block by clicking a button on the screen. They were instructed to judge the comparison sequence as slower, same, or faster than the standard sequence. To indicate their response, participants used the left arrow, down arrow, and right arrow keys on the computer keyboard, which had been labeled appropriately. The response started the next trial after a delay of 2 s. The experiment lasted approximately 30 min. Figure 5 gives a schematic picture of the task.

### Results

#### Musicians

**Figure 6.**
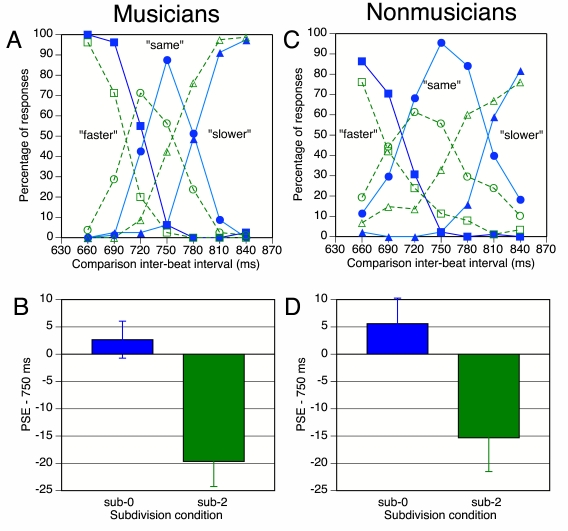
Results of Experiment 2b: (A, C) Mean response percentages as a function of subdivision condition and IBI duration of comparison sequences. Filled symbols and solid lines represent the sub-0 condition; empty symbols and dashed lines, the sub-2 condition. (B, D) Mean points of subjective equality (PSEs), expressed as deviations from the standard IBI duration. Error bars represent between-participant standard errors.

Figure 6a shows the mean percentages of “faster,” “same,” and “slower” responses as a function of subdivision condition and comparison IBI duration. It can be seen that relative to the sub-0 condition (solid lines), the response distributions in the sub-2 condition (dashed lines) were shifted to the left. This implies that a subdivided comparison sequence had to be faster than the simple standard sequence to be judged as having the same tempo, as predicted by the FDI hypothesis.

To test the reliability of this shift, each participant’s mean point of subjective equality (PSE) was computed for each subdivision condition. The PSE represents a weighted mean of the comparison IBI durations, with the number of “same” responses serving as weights.

Figure 6b shows the mean PSEs as deviations from 750 ms, the standard IBI duration. It can be seen that the PSE in the sub-0 condition was close to zero, whereas in the sub-2 condition the subdivided comparison sequence IBI had to be 19.6 ms shorter (2.6% faster) on average than the standard sequence to sound equal in tempo. This PSE is clearly different from zero, *t*(9) = 4.27, *p* = .001, and it is also significantly different from the PSE in the sub-0 condition, *t*(9) = 3.96, *p* < .005 (two-tailed). Individual subdivision effects (the difference between the PSEs in the sub-0 and sub-2 conditions as a percentage of the PSE in the sub-0 condition) ranged from -0.6% (the harpist) to 6.1% (author M.B.). A positive percentage here represents an effect in the expected direction.

The significance of individual PSEs could not be tested easily, but their correlations with previous results could be computed. The correlation with the sub-2 effect in reproduction (Experiment 2a) was significant, *r*(8) = .71, *p* < .05, but the correlations with the sub-1 and sub-2 effects in synchronization-continuation (Experiment 1) were small and not significant. Omission of the outlier clarinetist increased all correlations (.77, .51, and .51, respectively), but still only the correlation with reproduction was significant (*p* < .05).

#### Nonmusicians

Nonmusicians clearly found the task more difficult than musicians. One participant’s data were excluded because they appeared to be quite random, even in the sub-0 condition. Nonmusicians gave more “same” responses overall, and their response functions were less steep than those of the musicians (Figure 6c). However, their response functions did exhibit a leftward shift in the sub-2 condition, similar to that shown by the musicians. This is confirmed by the mean PSEs in Figure 6d. The mean PSE in the sub-2 condition was significantly different from zero, *t*(10) = 2.48, *p* = .033, and the difference between the sub-0 and sub-2 mean PSEs was very reliable, *t*(10) = 3.76, *p* = .004 (two-tailed). Individual effects ranged from -1.2% to 5.7%. However, there was no significant correlation with the individual subdivision effects in Experiment 2a, *r*(9) = .25.

A mixed-model ANOVA on the combined PSE data of musicians and nonmusicians revealed a highly reliable effect of subdivision condition, *F*(1, 19) = 29.6, *p* < .001, but no interaction with group. Thus, music training had no effect on the PSE.

### Discussion

 The predicted effect was obtained: A subdivided comparison sequence had to be faster than a simple standard sequence to be judged as having the same beat tempo. The average magnitude of the effect is similar to that obtained by Repp (2008) for musicians in sub-2 and sub-3 conditions. In contrast to the previous results, however, nonmusicians did not show smaller effects than musicians. The apparent dissociation between perception and reproduction found by Repp in a small group of nonmusicians may have been a fluke, but note that the present nonmusicians did not show a significant correlation between their results in the two tasks, whereas musicians did. 

## INTERLUDE: Some performance measurements

Experiments 1 and 2, being a replication of Repp (2008) with reversed roles of simple and subdivided sequences, were motivated by a desire to balance the overall design and to create a situation that is more similar to real music, where passages with higher note density (subdivisions of the beat) more often follow simple passages than the reverse. This order is most obvious with compositions in variation form, where a relatively simple theme is being elaborated in the following variations. Can the present findings, obtained with very primitive materials, be generalized to real music performance? The FDI hypothesis predicts that musicians should play variations of a theme slightly faster than the theme in order to compensate for the FDI and perceive themselves as playing at a constant tempo.

We first explored this hypothesis in a very informal and preliminary way by measuring the tempo of the theme and first variation in commercial recordings of two Beethoven piano sonatas that contain movements in variation form. The scores of these sonatas do not indicate any tempo change between the theme and the first variation. Of course, an artist might decide that a tempo change is nevertheless appropriate for expressive reasons; therefore, as long as the artist’s intentions are not known, performance measurements cannot provide conclusive evidence in favor of the FDI hypothesis. Nevertheless, a tendency to accelerate slightly and imperceptibly during the first variation would be consistent with the FDI hypothesis, whereas strict maintenance of the tempo or a tendency to slow down would contradict the hypothesis. A more substantial and clearly noticeable acceleration (by more than 5%, say), while compatible with the FDI hypothesis, would suggest a conscious artistic choice of a faster tempo.

### Methods

**Figure A1.**
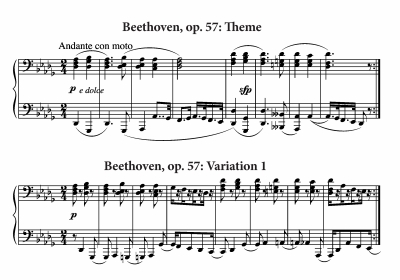
The first eight bars of the theme and of the first variation in the second movement of Beethoven’s Sonata in F minor, op. 57.

**Figure A2.**
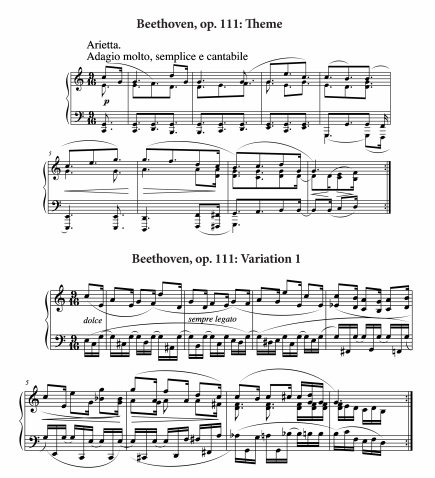
The first eight bars of the theme and of the first variation in the second movement of Beethoven’s Sonata in C minor, op. 111.

The music selected was the second movement of Beethoven’s *Appassionata* Sonata in F minor, op. 57, and the second movement of his final Piano Sonata in C minor, op. 111. Both movements consist of a 16-bar theme followed by a number of variations.Figures A1 and A2 in the Appendix show the first eight bars of the theme and the first eight bars of the first variation of each movement. Although neither theme consists entirely of simple beats (especially in op. 57 there is considerable rhythmic variation), the note density is clearly sparser than in the subsequent variation, which consists entirely of subdivided beats in each case. In op. 57, the subdivision is duple (sub-1) or quadruple (sub-3); in op. 111, it is triple (sub-2).

Twenty-eight recordings of op. 57 and 32 recordings of op. 111 were measured. Most of the recordings were CDs as well as a few LPs housed in the Yale Music Library; the remainder came from B.H.R.’s private collection and included some taped radio broadcasts. A listing of the recordings and the measurements can be found in Tables A1 and A2 in the Appendix.

The measurements were performed by B.H.R. using the second hand of his wristwatch. Maximum accuracy was not considered necessary in this preliminary exploration, and measurement errors of ±1 s may have occurred. The total durations of the first eight bars of the theme and of the first eight bars of the first variation (as shown in Figures A1 and A2) were measured by noting down the time to the nearest second at the initial downbeat and at the first downbeat of the repeat, and then taking the difference. (Each eight-bar section is repeated in performance.) The mean IBI was then obtained by dividing the duration by the number of beats (16 in op. 57; 24 in op. 111).

### Results

**Figure 7.**
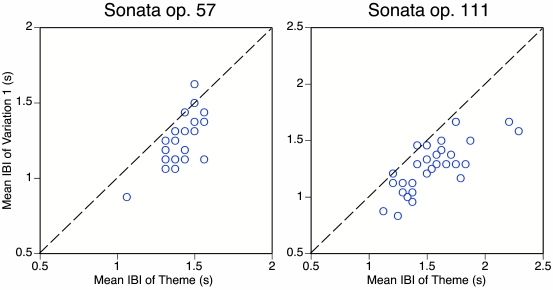
Mean inter-beat interval (IBI) of the theme plotted against the mean IBI of the first variation for two Beethoven sonata movements. Some data points coincide. The diagonal line indicates equality.

In Figure 7, the mean IBIs of the theme and the first variation are plotted against each other for each Beethoven sonata. Each data point corresponds to at least one performance, as some data points coincide. All data points falling below the diagonal line indicate that the variation was played faster than the theme. This was the case for 24 of 28 performances of op. 57 (three showed no difference, one a slowing down) and for 29 of 31 performances of op. 111 (one showed no difference, one a slowing down). No statistical tests are needed to confirm that there is an overwhelming tendency among famous pianists to play the first variation faster than the theme.^13^

### Discussion

The acceleration in the first variation was often much greater than the modest change predicted by the FDI hypothesis. The individual changes in IBIs ranged from -8.3% to 28% in op. 57, and from -2.9% to 34.9% in op. 111. (A positive percentage represents an effect in the predicted direction.) The larger tempo changes are easily perceptible, which makes it likely that the artists themselves were aware of them. Therefore, most of the observed accelerations presumably reflect more or less conscious artistic decisions, not the automatic adjustment predicted by the FDI hypothesis. In other words, most pianists simply did not intend to maintain the tempo of the theme, even though there are no indications in the score that the tempo should change.

One of the most famous interpreters of Beethoven’s sonatas, Artur Schnabel, is on record as having said, “The feeling of one central tempo for the entire work must be maintained, especially when a composer increases the motion from one variation to the next, as Beethoven does both in the *Appassionata*, op. 57, and in the Sonata in C minor, op. 111. Their point is lost if the speed changes at all.” (cited in Wolff, 1972, p. 79). Winter (1990), too, emphasizes the importance of maintaining a steady basic pulse in the second movement of op. 111. Paradoxically, Schnabel shows the largest acceleration of all pianists in op. 111 (34.9%) and the second-largest in op. 57 (22.7%). It is inconceivable that he was unaware of these large tempo changes. It can only be concluded that he considered himself exempt from following his own teachings. Winter attributes the similar tendencies of many other pianists to the influence of Schnabel’s seminal recordings, going so far as to call one performance a “caricatured imitation” of Schnabel. It is quite possible, however, that different artists converged independently on similar interpretive solutions (even though all must have been familiar with Schnabel’s path-breaking recordings, made in the 1930s).

Some pianists showed only small accelerations (< 5%), such as would be predicted by the FDI hypothesis. In op. 57, these pianists include Emil Gilels, Yves Nat, Maurizio Pollini, and Artur Rubinstein; in op. 111, Friedrich Gulda (in one recording), Maurizio Pollini, Rudolf Serkin, and Solomon. At least some of these pianists (Gulda, Pollini, Serkin, Solomon), on the basis of their general reputation and style of playing, could plausibly be regarded as “literalists” who may have tried to adhere closely to the score and thus may have intended to keep a constant tempo. If so, they might be the ones who show pure compensation for the FDI.

Apart from compensation for the FDI, it might be asked why there is such a strong general tendency to accelerate in the first variation, rather than to slow down. It seems to be common knowledge among musicians (cf. the Karajan quotation in the epigraph) that there is a tendency to accelerate when the music gets busier. Although this tendency is often considered as something to be avoided, the present measurements suggest that many distinguished artists nevertheless give in to it. This implies that the tendency is sometimes judged to be artistically acceptable and musically appropriate, at least in the contexts considered here. Perhaps, busier music often needs some “help” from the performer to acquire the appropriate character of forward motion. The FDI may lie at the origin of this tendency. Studies of expressive timing have suggested that the large rubato observed in performances of certain Romantic compositions is largely an amplification of smaller obligatory timing variations that are induced by the musical structure and that are reflected in perception of timing as well (Penel & Drake, 1998; Repp, 1998, 1999). Similarly, the tendency to speed up in busier music may be a more or less intentional amplification of an obligatory tendency fomented by the FDI.^14^

## EXPERIMENT 3a

Although the foregoing performance data are by no means irrelevant to the FDI hypothesis, it is difficult to draw conclusions from performances when it is not known whether the artist intended to maintain a constant tempo. Experiment 3a investigated performances of much simpler music, consisting of a theme and three variations, in the laboratory. Participants were instructed not to change the tempo. The FDI hypothesis predicts that an acceleration of tempo should nevertheless occur in the variations relative to the theme.

### Methods

#### Participants

Eight of the nine musician participants had served in Experiments 1 and 2. Two of the earlier participants (the cellist and author M.B.) were no longer available, but a third pianist joined the group. All but one of the nonpianists (a clarinetist) had had substantial piano training in addition to training on their primary instrument. About 5 months had elapsed since Experiments 1 and 2.

#### Materials

**Figure 8.**
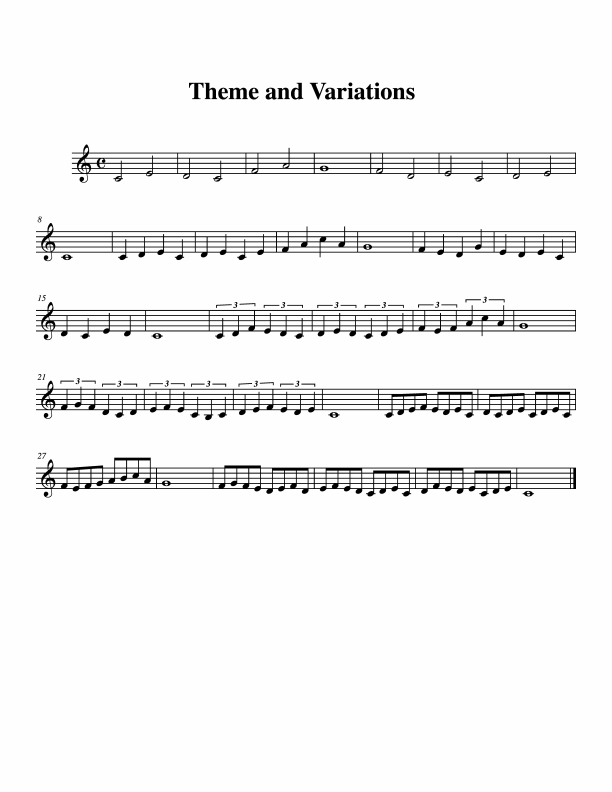
Theme and three variations, performed in Experiment 3a.

A simple theme with three variations was composed by author B.H.R. and is shown in Figure 8. Each segment consists of two four-bar phrases that end with a whole note. The theme proceeds in half notes (sub-0), the first variation in quarter notes (sub-1), the second variation in quarter-note triplets (sub-2), and the third variation in eighth notes (sub-3).

#### Procedure

The participants were told that this was a test of their ability to keep a constant tempo. Each participant was presented with the printed music and asked to look it over and rehearse it briefly, if necessary. Subsequently, they were asked to perform it three times on the Roland RD-250s digital piano, with short pauses between takes. The performances were recorded as MIDI text files. The single participant who had not had any piano lessons played the music (transposed) on his clarinet and was recorded via the computer microphone into an audio file. Before the first performance, and in some cases before each performance, a suggested tempo (80 half notes per minute) was given with an electronic metronome. If any error occurred, the performance was repeated. Participants were told neither to speed up nor to slow down but to play at the same tempo throughout.

### Results

**Figure 9.**
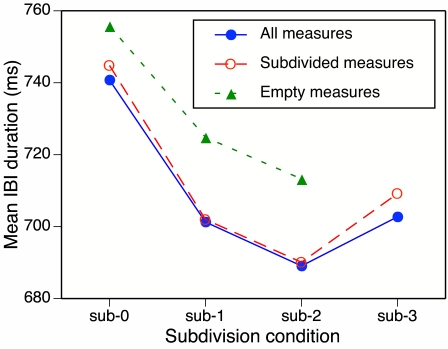
Results of Experiment 3a: Mean half-note inter-beat interval (IBI) duration in performances of the theme (sub-0) and three variations (sub-1, sub-2, sub-3), for all measures and separately for subdivided and empty measures (sub-3 omitted).

One pianist slowed down substantially after the theme in the first take only. This anomalous performance was omitted from analysis as an outlier. For each performance, IBIs were calculated from the recorded note onsets corresponding to half-note beats; whole-note intervals were divided by 2. (The final whole-note interval of the third variation was undefined because there was no following note onset.) The mean IBI for each segment (theme and three variations) was then calculated and averaged across the three takes.^15^ Figure 9 shows these mean IBIs averaged across participants (filled circles). Error bars are not included because they reflect only overall tempo differences across participants, which are not of interest. (Some participants deviated considerably from the suggested tempo.)

The mean IBI of the theme (740 ms) is close to the suggested tempo of 80 beats (half notes) per minute (IBI = 750 ms). The three variations were clearly played faster, however, as predicted by the FDI hypothesis. Their IBIs are 5-7% shorter, and their tempi amount to 86-87 beats per minute. A repeated-measures ANOVA comparing the theme against the variations revealed a significant difference, *F*(1, 8) = 9.0, *p* = .017. A second ANOVA comparing just the three variations with each other did not show any significant difference, *F*(2, 16) = 0.7, *p* = .455.

The mean data are representative of six of the nine participants. Two participants (the clarinetist who had shown anomalous results in Experiments 1 and 2, and the harpist) sped up for the first variation but then slowed down for the second and/or third variation. Another participant (the pianist whose first take was discarded as an outlier) slowed down for the first and second variations but then sped up somewhat.

Subdivided measures make up only 75% of each segment of the composition; the remaining 25% consist of whole notes. According to the FDI hypothesis, the whole-note measures should have been relatively longer in duration than the surrounding measures because they are entirely empty. To address this issue, the mean half-note IBI durations were calculated separately for subdivided and empty (whole-note) measures. The data of the participant who played on the clarinet had to be omitted here because individual tone onsets could not be measured with sufficient accuracy in the audio waveform. The data for the first and second whole notes in the theme and the first two variations were first compared by eye because it seemed possible that the second, segment-final note would be played longer than the first, merely phrase-final note. Their durations were similar, however, and so they were averaged. The data from the third variation were not considered because of the undefined interval at the end. The results are shown in Figure 9 (open circles and triangles).

A repeated-measures ANOVA with the variables of subdivision condition (sub-0, sub-1, sub-2) and measure type (subdivided vs. empty) was conducted on these data. Although there was indeed a tendency for empty measures to be longer than subdivided measures, the main effect of interval type was not significant, *F*(1, 7) = 3.2, *p* = .116. The main effect of subdivision condition was significant, *F*(2, 14) = 8.2, *p* = .013, but the interaction was not, *F*(2, 14) = 1.4, *p* = .277. It can be seen in the figure that empty measures (whole notes) were affected by subdivision of surrounding half-note IBIs nearly as much as were the half-note IBIs themselves.

### Discussion

The results of this experiment demonstrate that an effect of metrical subdivision on musical performance tempo can be obtained in highly trained musicians who intend to keep the tempo constant. This extends the earlier finger-tapping findings to a situation that is closer to realistic music performance. The mean subdivision effect was actually a bit larger than in the earlier experiments, and therefore it is not unreasonable to expect the effect to grow even larger in more complex music that is played with more expressive freedom, such as the Beethoven sonata excerpts measured in the Interlude.

The music composed for this experiment was simple and easy to play. Even those musicians whose primary instrument was not the piano should not have encountered any technical difficulties. Therefore, it is interesting to note that the number of subdivisions had no effect: The acceleration relative to the theme (sub-0) was the same in all three variations (sub-1, sub-2, and sub-3). Some previous experiments in this series (Repp, 2008) have found an effect of number of subdivisions. Perhaps the order of subdivision conditions needs to be varied to find such effects. This warrants further investigation.

A novel finding of this experiment is that the effect of subdivision spreads to contextual intervals that are not subdivided. This may have been due to mental subdivision of the empty intervals. Indeed, there was no clear evidence for any strictly local effect of subdivision in this experiment. It is also true, however, that the majority (75%) of the intervals in the variations were subdivided. Perhaps, evidence of a local subdivision effect could be found in materials in which a minority of intervals is subdivided. This was investigated in the next two experiments.

## EXPERIMENT 3b

Experiment 3b, like Experiment 3a, involved elementary music performance. In the materials, only one out of every three IBIs was subdivided. Would these intervals be played faster than the surrounding intervals, and/or would there still be a global effect of subdivision, such that the whole sequence would be played faster than a sequence not containing any subdivisions?

### Methods

#### Participants

The participants were the same as in Experiment 3a. Experiment 3b followed immediately in the same session.

#### Materials

**Figure 10.**
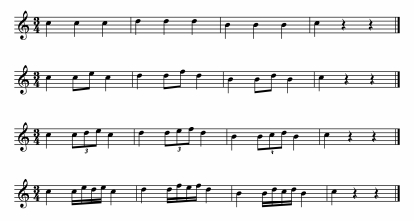
Musical materials for Experiments 3b and 3c.

Four simple melodies, which also can be considered a theme with three variations, were composed for this experiment. They are shown in Figure 10. Each melody consists of four three-beat measures, in the last of which only the downbeat is marked by a tone onset. In the first three measures, each beat is marked by a tone onset, and while the first and third IBIs are always simple, the second IBI is either simple (sub-0) or subdivided (sub-1, sub-2, or sub-3).

#### Procedure

Participants were given the music sheet and were asked to play the set of four melodies three times, without interruption. Before they started, a suggested tempo of 84 quarter-note beats per minute (IBI = 714 ms) was given with a metronome. The importance of keeping a constant tempo was stressed.

### Results

**Figure 11.**
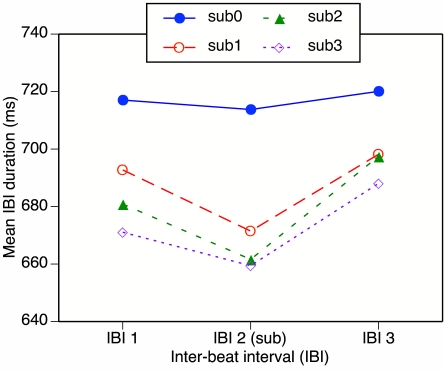
Results of Experiment 3b: Mean within-measure inter-beat intervals (IBIs) in the melodies as a function of position in the measure and subdivision (sub) condition.

The tone onsets in the audio recording of the clarinetist proved too difficult to measure accurately; only the MIDI recordings from the piano could be used. The quarter-note IBIs were determined for the first three measures of each melody and then averaged across the three measures and the three repetitions. Figure 11 shows these mean IBIs averaged across participants. Error bars are omitted, for reasons stated earlier.

In the baseline (sub-0) condition participants played close to the suggested tempo, and there was little difference in IBI duration as a function of position in the measure. In the sub-1, sub-2, and sub-3 conditions, however, the playing tempo was clearly faster and tended to increase with the number of subdivisions. Moreover, the subdivided second IBI tended to be shorter than the preceding and following simple IBIs.

The data were subjected to a repeated-measures ANOVA with the variables of subdivision condition and IBI position. Both main effects were highly reliable, *F*(3, 21) = 11.7, *p* = .001, and *F*(2, 14) = 14.4, *p* = .001, respectively. However, the interaction fell short of significance, *F*(6, 42) = 2.6, *p* = .091. In separate comparisons of each subdivision condition with the baseline, the interaction did reach significance for sub-1, *F*(2, 14) = 6.0, *p* = .028, but was only close to significance for sub-2, *F*(2, 14) = 3.7, *p* = .073, and sub-3, *F*(2, 14) = 3.7, *p* = .056. In an ANOVA on the three subdivision conditions with the baseline omitted, the main effect of subdivision condition was not significant, *F*(2, 14) = 1.5, *p* = .261, nor was the interaction. (The main effect of IBI position remained significant, of course.) Thus, the results for the three subdivision conditions must be considered statistically equivalent.

Each melody also included an empty measure at the end, equivalent in duration to three IBIs. Because the melodies were played without interruption, the durations of these intervals, divided by 3, could be compared to those of the simple IBIs in the other measures. (Only the duration of the very last empty measure was undefined.) Three participants made longer pauses between repetitions of the melody set (i.e., going from sub-3 back to sub-0), so the interval at the end of sub-3 was not considered further. A repeated-measures ANOVA with the variables of subdivision condition (sub-0, sub-1, sub-2) and measure type (empty vs. subdivided) was conducted on the data. There was no tendency to stretch the empty measures relative to the other measures, as the FDI hypothesis would predict, *F*(1, 7) = 1.3, *p* = .299. However, the main effect of subdivision was significant, *F*(2, 14) = 14.7, *p* = .001, and the interaction was not, *F*(2, 14) = 0.6, *p* = .508, which implies that the empty measures were affected by subdivision just as much as were the quarter-note IBIs, whether subdivided or not.

### Discussion

The results of this small performance study reveal that, even with only one out of three IBIs being subdivided, subdivision had a global accelerating effect on the timing of the whole melody, including adjacent simple IBIs and even the long melody-final intervals. Subdivision also had a local accelerating effect on the subdivided IBI relative to its simple IBI neighbors, as predicted by the FDI hypothesis, but that effect was smaller than the global effect.

Researchers at the Royal Technical University in Stockholm have developed rules for expressive computer music performance based on a musician’s intuitions and on perceptual evaluation (see, e.g., Sundberg, 1988). These rules include durational contrast: Notes (i.e., tones) with inter-onset interval (IOI) durations between 30 and 600 ms are shortened (and decreased in amplitude) relative to longer notes, according to a function with three linear segments. According to that function, shortening is greatest at 200 ms (16.5%) and decreases towards both shorter and longer durations (Friberg, 1991). This rule is consistent with the local effect of the FDI, but it predicts increasing effects of subdivision as the IOIs between subdivisions get shorter (up to 200 ms), which was not observed here, and it cannot account for the global effects found here. However, it is fair to note that the purpose of the durational contrast rule is to enhance expression, not to compensate for the FDI.

## EXPERIMENT 3c

In this final experiment, the materials of Experiment 3b were used in a perceptual judgment task that focused on the local effect of subdivision. According to the FDI hypothesis, subdivided IBIs will have to be shorter than simple IBIs in order for the beat tempo to be perceived as perfectly regular.

### Methods

#### Participants

They were the same as in Experiments 3a and 3b. Experiment 3c followed in the same session.

#### Materials

The melodies were the same as in Experiment 3b (see Figure 10). They were now played back under computer control on the Roland RD-250s digital piano, one melody per trial. All tones had a nominal duration of 40 ms (i.e., articulation was staccato throughout) and the same MIDI velocity. Each melody was played in seven versions. One version had constant IBIs of 720 ms. The other six versions were obtained by changing the duration of the second IBI in each measure by ±3%, ±6%, and ±9%. At the same time, the duration of the two simple IBIs in each measure was changed in the opposite direction by half the percentage. Thus the total duration of the melody remained unchanged, and the durational contrast between subdivided and simple IBIs was ±4.5%, ±9%, and ±13.5% of 720 ms. Eight blocks of 28 trials each were presented. The order of trials was freshly randomized for each block.

#### Procedure

Participants were required to judge the temporal regularity of the melody on each trial. On the computer screen they saw the statement “The beats in this melody were timed evenly/unevenly” and clicked one of the two response alternatives with the mouse. The response triggered the next trial after a delay of 2 s. A trial could be repeated if necessary. There were short pauses between blocks. The experiment took about 40 min.

### Results

**Figure 12.**
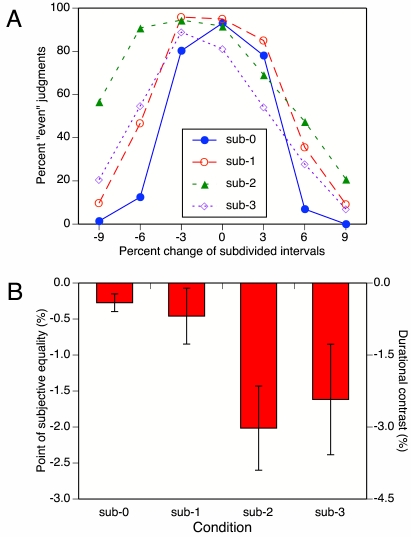
Results of Experiment 3c: (A) Mean percentages of “even” judgments as a function of percentage change of subdivided intervals (i.e., the second inter-beat interval, IBI, in each measure) and subdivision condition. (B) The corresponding mean points of subjective equality (PSEs), with between-participant standard error bars.

The mean percentages of “even” responses are shown in Figure 12a as a function of the percentage change of the second IBI in each measure and subdivision condition. One unexpected finding was that it was much more difficult to judge evenness of beat tempo when some IBIs were subdivided than when there were no subdivisions. With sub-0 melodies, participants could easily detect changes of ±6% or ±9%, though not changes of ±3%. In melodies containing subdivided IBIs, even the larger changes were often not detected, and the sub-2 condition (triple subdivision) was particularly difficult in that regard.

In addition, it can be seen that the response functions for the sub-2 and sub-3 conditions are asymmetric, with the peak shifted to the left, whereas the sub-1 function shows only a small shift. To quantify these shifts, PSEs were calculated, as in Experiment 2b. The mean PSEs are shown in Figure 12b. Whereas the sub-1 PSE hardly differs from the sub-0 PSE, the PSEs for the sub-2 and sub-3 conditions indicate that the subdivided IBIs had to be 2.5% to 3% shorter than the adjacent simple IBIs for the tempo to be judged maximally even.

A repeated-measures ANOVA on the PSEs showed that the main effect of subdivision condition fell just short of significance, *F*(3, 24) = 3.6, *p* = .057. Only six of the nine participants showed consistently negative PSEs in the sub-2 and sub-3 conditions. One (the harpist) showed positive PSEs in both conditions; one (the clarinetist who had shown anomalous effects in Experiments 1 and 2) showed a negative sub-2 PSE but a positive sub-3 PSE; and author B.H.R. showed very small PSEs, perhaps as a result of having been exposed to the stimuli during repeated pilot testing.

### Discussion

Because of the just mentioned individual differences, the PSE results of this experiment are not impressive, but the majority of participants did show the local subdivision effect predicted by the FDI hypothesis. For them, the subdivided IBIs had to be shorter than the simple IBIs for the beat tempo to sound maximally even.

Perhaps the more interesting result of this experiment is the difficulty of judging evenness of beat tempo in all melodies that contained subdivided IBIs. This finding implies considerable perceptual tolerance for either compression or expansion of subdivided IBIs, either of which may occur in the course of expressive music performance. Such expressive deviations thus are not likely to be perceived as irregularities of beat tempo, although they may affect the perceived character of a performance.

## GENERAL DISCUSSION

The purpose of this study was to extend previous findings by showing that continuous metrical subdivision of IBIs creates the impression of longer IBIs and hence leads to a compensatory acceleration of beat tempo in tapping, and by furthermore including situations involving actual music performance and materials with local subdivision. Experiments 1 and 2 used the same three tasks as Repp (2008) — synchronization-continuation tapping, beat reproduction by tapping, and perceptual judgment — but with reversed roles of simple and subdivided sequences, the latter always occurring in second position in a trial. On the whole, the results are consistent with the earlier findings, with some exceptions. Thus, the effect of subdivision in a rhythmic musical context does not seem to depend on the order in which simple and subdivided sequences are presented.

We argued that a simple beat followed by a subdivided beat is more common in real music than the reverse, particularly in compositions in variation form. In the Interlude we showed that concert artists tend to play continuously subdivided variations faster than a theme containing few subdivisions, and we confirmed this tendency in Experiment 3a, where we could be sure that the participating musicians really intended to maintain a constant tempo. Experiments 3b and 3c employed materials in which only one out of every three IBIs was subdivided, and we found that the subdivided IBIs tended to be played faster and judged to be relatively longer.

All these findings had been predicted by the FDI hypothesis, according to which subdivided intervals are subject to the filled duration illusion, demonstrated previously in various psychophysical studies. The present findings, together with those of Repp (2008), extend this phenomenon to musical contexts in which subdivisions are metrically regular and a beat tempo must be maintained. Moreover, the results show that even highly trained musicians are subject to this illusion. To be sure, the FDI found here is smaller than that reported in most psychophysical studies; this can be attributed to the metrical context and the expertise of the participants.

Why does the FDI occur? The present study was not specifically concerned with this question, but two possible explanations should be mentioned. According to one hypothesis, continuous or intermittent auditory input during an interval accelerates an internal pacemaker that emits pulses that are transmitted to an accumulator that measures interval duration in terms of pulse counts (Gibbon, Church, & Meck, 1984; Penney, Gibbon, & Meck, 2000). Wearden et al. (2007) found specific support for this hypothesis in a study comparing completely filled with empty intervals across a wide range of durations. According to the other hypothesis, which applies only to discrete subdivisions, processing of intervals takes a fixed time, and this time is added to the subjective duration of the interval (Nakajima, 1979, 1987). Each sub-interval of a subdivided interval (presumably, only down to some lower limit of duration) exerts a temporal processing cost, so that the subjective duration of the whole interval increases by a multiple of the fixed processing time. According to Nakajima, this time is about 80 ms, but this value was estimated from psychophysical studies with single intervals and clearly is too large for the present contexts. Even with a smaller increment, one might expect more consistent increases in the FDI with the number of subdivisions than were obtained in the present research, but it is likely that there is a lower limit to interval durations that can be processed independently, perhaps around 200 to 250 ms (see Repp, 2003). Therefore, the effect of subdivision may quickly reach an asymptote as the number of subdivisions is increased.

Experiment 3 yielded two interesting findings that had not been predicted. One is that subdivision of some IBIs but not others in a musical sequence affects the global performance tempo. This was true regardless of whether the subdivided IBIs constituted 75% or only 33% of the sequence, and this global effect (an acceleration relative to a sequence of simple beats) was larger than the difference between adjacent simple and subdivided IBIs. This result goes beyond what the FDI hypothesis predicts and suggests that the effect of subdivision is not restricted to the subdivided interval but spreads to contextual intervals. This, incidentally, is more compatible with a pacemaker hypothesis than with Nakajima’s (1987) temporal processing hypothesis, for an accelerated pacemaker could easily remain accelerated for several seconds, whereas interval processing costs are strictly local. However, it is also consistent with the possibility of mental subdivision strategies that are induced by the subdivided beats. The second novel finding is that the unevenness of beat tempo is more difficult to detect in sequences that contain a mixture of simple and subdivided IBIs than in simple beat sequences (and, presumably, than in continuously subdivided sequences). In other words, listeners can tolerate considerable amounts of expansion or compression of subdivided IBIs without perceiving any temporal irregularity. This opens the door to expressive timing in performance, which usually does not interfere with the perceived stability of beat tempo.

The difficulty of detecting unevenness in mixed sequences was particularly evident with triple subdivision (sub-2). This adds to previous evidence that triple subdivision is often more difficult than duple or quadruple subdivision (Bergeson & Trehub, 2006; Bolton, 1894; Drake, 1993; Repp, 2003, 2007). The deviant results for triple subdivision in Experiment 1 point in the same direction, although their precise cause remains unclear.

In each experiment there were considerable individual differences, even though participants (except for the two authors) were comparable in terms of musical training and experience. Two in particular stood out. The clarinetist who showed a reversed subdivision effect in Experiment 1 and a partially reversed effect in Experiment 2a was the only participant who had extensive experience in synchronization tasks; she also had been a participant in Repp (2008). Although she presumably tried to follow instructions, she was not naïve and perhaps involuntarily counteracted the subdivision effect. The other participant was the harpist, who seemed to be rather consistently immune to subdivision effects. She has been observed informally to engage frequently (in this study and others as well) in subdivision of the beat by means of lip and head movements. It is possible that this habit helped her overcome the effect of physical IBI subdivision. The majority of participants, however, did not seem to have used mental subdivision strategies; or, if they did, the strategies were not sufficiently effective to cancel the effects of physical subdivision.

Spontaneous subdivision strategies, whether overt or covert, were not controlled in this study or in Repp (2008). This raises some interesting questions for future research: Can IBI subdivision that is carried out solely by the participant, using movement or mental imagery, create a subdivision effect, and can it completely eliminate the effect of physical (auditory) subdivision? Wohlschläger and Koch (2000) found that subdividing sequence IBIs (and inter-tap intervals) with silent finger or toe movements reduced the negative mean asynchrony in synchronization, apparently due to a subjective lengthening of the sequence IBIs. Repp (2008, Experiment 2) showed that subdivision of sequence IBIs with additional taps during synchronization can generate a subdivision effect in continuation tapping, and that subdivision of inter-tap intervals during continuation tapping can neutralize the effect of physical subdivision during synchronization. However, the taps generated auditory feedback (either thuds or tones) and thus had physical consequences. The nature of sensory feedback from subdivisions is a variable to be considered in future studies.

The implications of the present findings for music performance may be more far-reaching than was first thought (Repp, 2008). Initial findings merely suggested that musicians would have to play music containing more notes slightly (unnoticeably) faster in order to compensate for the subjective slowing of such passages. However, the results of Experiment 3a and particularly the measurements of Beethoven sonata performances indicate that accelerations in real performance are often much larger than would be required to compensate for the FDI. The FDI thus may merely be the germ of a more pervasive tendency to accelerate in dense passages, which musicians sometimes try to avoid but at other times seem to follow quite happily, probably because they find it expressively appropriate. A simple and intuitively reasonable argument, compatible with the pacemaker acceleration hypothesis, is that a high auditory event rate increases arousal (Balch & Lewis, 1999; Husain, Thompson, & Schellenberg, 2002), and that musicians want to communicate their heightened excitement to listeners, thereby exaggerating the subdivision effect that would be predicted by the FDI alone.

## Footnotes

1 In this epigraph, the famous conductor Herbert von Karajan recalls one of several laboratory experiments in which he participated (see Wagner, 1974). The quotation should be taken with a grain of salt because we cannot find any experiment in Wagner’s article that matches Karajan’s description, which is not very precise to begin with. Nevertheless, we cite it here because of its relevance to the present research, if we have interpreted it correctly. We think Karajan is saying that, when asked to play with increasingly smaller note values while trying to maintain a constant beat tempo, he was quite accurate but nevertheless accelerated by 2-3%.

2 These two types of the FDI may well have different causes. Repp and Marcus (submitted) have failed to find an FDI due to continuous sound in tasks very similar to those in the present Experiments 1 and 2.

3 The perceptual underestimation hypothesis of Wohlschläger and Koch (2000) predicts that participants should tap faster when continuing a simple beat, but this prediction was not confirmed in Repp’s (2008) study, nor did subdivision reduce the negative mean asynchrony during synchronization. The FDI hypothesis is neutral with regard to the absolute produced or perceived tempo; it only concerns the difference between filled and empty intervals.

4 The data were unclear as to whether the effect of subdivision is constant or proportional to IBI duration.

5 If the computer produced the subdivisions, they would function as pacing stimuli and thus would influence the timing of the participant’s beat taps.

6 The event onsets we refer to as beats in this study plausibly functioned as the main beat (tactus) of the stimulus sequences. Although slower or faster main beats are conceivable, such differences in subjective metrical organization are probably irrelevant to the FDI.

7 This was a difference from the baseline condition in Experiment 2 of Repp (2008), which did not include feedback tones. The tones were delayed by about 15 ms, due to electronic processing. This small delay was not noticeable and was not expected to affect performance.

8 See Repp (2008, Experiment 1) for reasons to omit the first two continuation intervals and for evidence of tempo stability in continuation tapping of a comparable group of musicians.

9 The Greenhouse-Geisser correction was applied to all F values in this study that have more than one degree of freedom in the numerator.

10 With this participant omitted, the main effect of subdivision was also significant in the overall ANOVA, *F*(2, 16) = 10.436, *p* = .001.

11 When we designed this experiment for musicians, we did not yet know the results of Experiment 1. Had we known them, we might have been reluctant to choose the sub-2 condition. As for the nonmusicians, they were run at a later date; they had not been part of the original design, hence the mismatch in conditions.

12 We considered this cuing method preferable that of Experiment 1 because auditory cues might induce mental subdivision of a subsequent undivided beat, a strategy that might counteract the effect we were looking for.

13 Measurements of the theme and all variations in op. 111 for a smaller sample of 15 performances appear in Table 2.1 of Winter (1990). Only 6 of them are contained in the present sample; 9 are not. Nevertheless, the conclusions are the same: 13 out of 15 pianists in Winter’s sample played the first variation faster than the theme.

14 In the summer of 2008, author B.H.R. had the opportunity to hear the distinguished Canadian pianist Marc-André Hamelin in concert. Throughout his performance of Mozart’s Sonata in A minor, K. 310, he clearly accelerated whenever there were 16th notes in the score. Even though this tendency was unusually pronounced, it did not seem aesthetically objectionable (piquant, rather) and may serve as another example of a (deliberate or unconscious) expressive strategy related to the FDI.

15 There was a clear tendency to play faster in the later takes, but because the experimenter (author B.H.R.) had been inconsistent in giving metronome cues between performances, these differences are not reported or interpreted.

## Appendix A

**Table A1.** Recordings of Beethoven’s Piano Sonata in F Minor, op. 57, Measured in This Study.

Pianist	Recording	Mean beat duration (s)			Mean tempo (bps)	
		Theme	Var1	diff (%)	Theme	Var1
Ashkenazy	SXL 6603 or SXL 6994^a^	24	22	8,3	40	44
Backhaus	Decca 433 882-2	23	18	21,7	42	53
Brendel	Philips 412 586-2	24	21	12,5	40	46
Cliburn	RCA Victor 60356-2-RG × TOT	21	17	19,0	46	56
Fischer, A.	Hungaroton HCD 31631	25	18	28,0	38	53
Fischer, E.	Pearl GEMM CD 9218	22	20	9,1	44	48
Frank	Music & Arts CD 640	24	24	0,0	40	40
Gieseking 1	VAI VAIA 1088	25	23	8,0	38	42
Gieseking 2	M & A UPC#0-17685-10742 9	23	23	0,0	42	42
Gieseking 3	EMI 5 67585 2	23	23	0,0	42	42
Gilels	Brilliant Classics 92132/6	22	21	4,5	44	46
Gulda 1	Decca 475 6835	21	19	9,5	46	51
Gulda 2	Brilliant Classics 92773	21	18	14,3	46	53
Horowitz 1	RCA Victor 09026-68977-2	21	18	14,3	46	53
Horowitz 2	Sony SK 53467	22	18	18,2	44	53
Kempff	The 50s CD 14 B	23	19	17,4	42	51
Lamond	Biddulph LHW 043	17	14	17,6	56	69
Levy	Marston 52021-2	25	22	12,0	38	44
Medtner	Appian APR 5546	22	18	18,2	44	53
Moravec	VAI VAIA 1069	23	19	17,4	42	51
Nat	EMI CDZ 7 62907 2	21	20	4,8	46	48
Pollini	DG 474 451-2	22	21	4,5	44	46
Richter 1	Praga PR 254 021	21	19	9,5	46	51
Richter 2	RCA Victor 07863-56518-2	22	18	18,2	44	53
Rubinstein	RCA Red Seal 74321 68006 2	22	21	4,5	44	46
Schnabel	EMI CDH 7 63770 2	22	17	22,7	44	56
Solomon	Testament SBT 1192	24	26	-8,3	40	37
Watts	EMI CDC-7 49264 2	23	21	8,7	42	46

*Note*. Measurements are for the initial eight bars of the theme and of the first variation (Var1).

a Taped from the radio.

**Table A2.** Recordings of Beethoven’s Piano Sonata in C Minor, op. 111, Measured in This Study.

Pianist	Recording	Mean beat duration (s)			Mean tempo (bps)	
		Theme	Var1	diff (%)	Theme	Var1
Ashkenazy 1	Berlin Classics BC 2133-2	42	31	26,2	34	46
Ashkenazy 2	(Live, Salzburg Festival 1981) a	36	32	11,1	40	45
Backhaus 1	(Live, Salzburg Festival 1966) a	30	20	33,3	48	72
Backhaus 2	Decca 433 882-2	27	21	22,2	53	69
Brendel	Philips 412 589-2	38	33	13,2	38	44
Fischer, A.	Hungaroton HCD 31628	33	25	24,2	44	58
Fischer, E.	Music & Arts CD 880	31	27	12,9	46	53
Frank	Music & Arts CD 640	39	34	12,8	37	42
Gieseking	Music & Arts CD 743	29	27	6,9	50	53
Goode	Nonesuch 0 79211-2	41	33	19,5	35	44
Gould	Columbia ML 5130	45	36	20,0	32	40
Gulda 1	(Live, Salzburg Festival 1964) a	36	29	19,4	40	50
Gulda 2	Brilliant Classics 92773	36	35	2,8	40	41
Guller	Nimbus NI 5061	34	31	8,8	42	46
Horszowski	Vox Legends CDX2 5500	39	36	7,7	37	40
Hungerford	Vanguard Classics OVC 5001	44	31	29,5	33	46
Kempff 1	The 50s- CD 14 B	33	23	30,3	44	63
Kempff 2	DG 453 010-2	31	25	19,4	46	58
Levy	Marston 52007-2	53	40	24,5	27	36
Marik	Arbiter 149	29	29	0,0	50	50
Nat	EMI CDZ 7 62907 2	32	24	25,0	45	60
Petri	Pearl GEM 0149	38	31	18,4	38	46
Pogorelich	DG 410 520-2	55	38	30,9	26	38
Pollini	DG 429 570-2	42	40	4,8	34	36
Richter 1	Music & Arts CD-1025	37	30	18,9	39	48
Richter 2	Brilliant Classics 92229	33	27	18,2	44	53
Rosen	Columbia M3X 30938	40	31	22,5	36	46
Schnabel	EMI CDH 7 63765 2	43	28	34,9	33	51
Serkin	Sony SM3K 64490	36	35	2,8	40	41
Solomon	Testament SBT 1188	42	40	4,8	34	36
Vogel	BBC MM119	34	35	-2,9	42	41

*Note*. Measurements are for the initial eight bars of the theme and of the first variation (Var1).

a Taped from the radio.
